# Positive Association between Triglyceride-Rich Lipoprotein Cholesterol and Diabetes Mellitus in Hypertensive Patients

**DOI:** 10.1155/2021/7722269

**Published:** 2021-12-01

**Authors:** Wei Zhou, Yu Yu, Lingjuan Zhu, Wangsheng Fang, Yu Tao, Minghui Li, Xiao Huang, Tao Wang, Huihui Bao, Xiaoshu Cheng

**Affiliations:** ^1^Center for Prevention and Treatment of Cardiovascular Diseases, The Second Affiliated Hospital of Nanchang University, Nanchang, Jiangxi, China; ^2^Department of Cardiovascular Medicine, The Second Affiliated Hospital of Nanchang University, Nanchang, Jiangxi, China; ^3^Cardiac Arrhythmia Center, Fuwai Hospital, National Center for Cardiovascular Diseases, Chinese Academy of Medical Sciences and Peking Union Medical College, Beijing, China; ^4^Wuyuan County Health Committee, Wuyuan, Jiangxi, China

## Abstract

**Background:**

The association between triglyceride-rich lipoprotein cholesterol (TRL-C) and diabetes mellitus (DM) remains unclear because of limited research and data. The aim of this study was to explore the independent association between TRL-C and DM in hypertensive patients and to examine whether a healthy lifestyle would have an impact on this relationship.

**Methods:**

In this study, data from 13,721 hypertensive patients who were not treated with lipid-lowering drugs were analyzed. TRL-C was calculated from total cholesterol (TC) minus [LDL cholesterol + HDL cholesterol]. DM was defined as fasting plasma glucose of ≥7.0 mmol/L and/or self-reported history of hypoglycemic drug use.

**Results:**

After adjusting for potential confounding factors, the TRL-C was significantly positively associated with elevated DM (odds ratio (OR): 1.73 and 95% confidence interval (CI): 1.54–1.94). In subgroup analysis, a healthy lifestyle (HL) failed to modify the positive association between TRL-C and DM (HL: OR 1.93, 95%CI 1.58–2.36; non-HL: OR 1.72, 95%CI 1.50–1.98; *P* for interaction = 0.38).

**Conclusion:**

The results showed a positive association between TRL-C and DM in hypertensive patients. A healthy lifestyle failed to diminish the relationship between TRL-C and DM. The novel findings indicate that TRL-C might be a reliable marker of DM and may provide a new strategy for the prevention and treatment of DM.

## 1. Introduction

Diabetes mellitus (DM) is one of the most common risk factors for cardiovascular disease (CVD), renal insufficiency, retinopathy, and all-cause mortality [1–3]. Clinical and basic research on exploring diabetes-related risk factors remains ongoing. Recently, researchers have focused on the important role of lipids in patients with diabetes [4, 5]. A large-scale study on screening lipid profiles associated with DM in China was conducted, and six lipids were finally identified to be significantly associated with DM [6]. However, after a careful review of this study, we found that there were still some lipids that were not explored.

Triglyceride-rich lipoprotein cholesterol (TRL-C), also known as remnant cholesterol, is an important risk factor for atherosclerosis [7]. Previous studies have found that TRL-C is closely related to atherosclerosis-related diseases, such as CVD, ischemic stroke (IS), and peripheral artery disease (PAD) [8, 9]. Recently, there has been increasing evidence suggesting there is an interrelationship between atherosclerosis and DM [10–12]. Therefore, TRL-C, as a risk factor for atherosclerosis, may also be closely associated with DM. However, large clinical studies for exploring the relationship between TRL-C and DM are absent. In addition, maintaining a healthy lifestyle, mainly including weight management, physical activity, smoking and alcohol cessation, and vegetable and fruit intake, is an important measure for the prevention and treatment of DM [13]. Therefore, a healthy lifestyle may affect the relationship between TRL-C and DM, and it is necessary to explore the relationship between TRL-C and DM under a healthy lifestyle.

Notably, the coexistence of DM and hypertension significantly increases the risk of CVD, stroke, nephropathy, and retinopathy [14]. Therefore, a better understanding of DM-related risk factors in hypertensive patients may reduce the enormous burden of DM and its associated complications. In order to solve the abovementioned problems, the present study aimed to examine the association between TRL-C and DM in hypertensive patients, as well as to explore the relationship between TRL-C and DM in a healthy lifestyle, by analyzing data from the China Hypertension Registry Study.

## 2. Materials and Methods

### 2.1. Study Design and Participants

The study data were drawn from the China Hypertension Registry Study (http://www.chictr.org.cn/, no: ChiCTR1800017274), and details about the purpose, protocol, and outcome of the study have been described [15]. The inclusion and exclusion criteria for the study are described in detail in Table S1. In short, this study is a large observational cohort study of patients with hypertension. The aim of the study was to explore the prevalence of hypertension in China and the risk factors that influence its treatment and prognosis. Hypertension was defined as systolic blood pressure (SBP) of values ≥ 140 mmHg and/or diastolic BP (DBP) of values ≥ 90 mmHg, self-reported history of hypertension, or the use of antihypertensive drug(s) at baseline [16]. From March to August 2018, a total of 14,268 patients with hypertension were recruited into our study in Wuyuan, Jiangxi Province, China. Finally, a total of 13,721 hypertensive patients who were untreated with lipid-lowering drugs were included in our study for analysis.

The study was conducted in accordance with the Declaration of Helsinki and was approved by the Ethics Committee of the Anhui Medical University Biomedical Institute (no. CH1059) [17], and all study participants signed informed consent.

### 2.2. Data Collection and Outcome Definition

All study participants were required to collect fasting, venous blood samples by trained study staff during the baseline data collection period. Total cholesterol (TC, mmol/L), triglycerides (TG, mmol/L), low-density lipoprotein cholesterol (LDL-C, mmol/L), high-density lipoprotein cholesterol (HDL-C, mmol/L), and fasting blood glucose (FBG, mmol/L) were measured by an automatic clinical analyzer (Beckman Coulter, USA) in Biaojia Biotechnology Laboratory, Shenzhen, China. Estimated glomerular filtration rate (eGFR, ml/min/1.73 m^2^) was calculated using the chronic kidney disease epidemiology collaborative (CKD-EPI) formula [18]. Non-HDL-C was calculated as HDL-C subtracted from TC [7]. TRL-C was defined as non-HDL-C minus LDL-C, which was estimated by the Friedewald formula as approximately TG/5 mmol/L [19].

Blood pressure (BP, mmHg) was measured by electronic sphygmomanometers after the subjects had rested for 10 min. Body mass index (BMI, kg/m^2^) was calculated by dividing weight by the square of height. Other covariates were obtained through questionnaires, including age, sex (male and female), physical activity (moderate or vigorous exercise, rarely exercise), fruit and vegetable consumption (≥500 g/day, <500 g/day), smoking status (never, former, and current), drinking status (never, former, and current), disease, and medication history (including self-reported stroke and CVD, antihypertensive drugs).

### 2.3. Definition of DM and Healthy Lifestyle Index

DM was defined as fasting plasma glucose of ≥7.0 mmol/L and/or self-reported history of hypoglycemic drug use [20]. The healthy lifestyle index was defined as the composition of five modifiable lifestyle factors, including BMI, physical activity, smoking status, drinking status, and fruit and vegetable consumption [21]. Regular exercise was defined as ≥150 minutes/week of moderate-intensity physical activity or ≥75 minutes of vigorous-intensity physical activity [22]. Score 1 point for each healthy lifestyle component, with the total score ranging from 0 to 5. A healthy lifestyle means that participants with a BMI <25 kg/m^2^, ≥150 minutes/week of moderate-intensity physical activity or ≥75 minutes/week of vigorous-intensity physical activity, nonsmoking, nondrinking, and fresh fruit and vegetable consumption of ≥500 g/day, which added up to 5 points [23]. Details of the healthy lifestyle score are described in Table S2.

### 2.4. Statistical Analysis

Baseline characteristics of the study population were presented according to with or without DM and the different groups were compared using ANOVA tests or Chi-square tests. Continuous variables are presented as the mean ± SD, and categorical variables are presented as percentage (%). Univariate analysis was to find out which factors are related to outcome variables and to provide a reference for controlling confounding factors in logistic regression analysis. Logistic regression analyses were performed to assess the independent association of TRL-C with elevated DM by presenting the odds ratio (OR) and 95% confidence interval (CI) after adjusting for confounding factors. A fully adjusted generalized additive model (GAM) and smoothing curve fitting (penalized spline method) visually demonstrated the relationship between TRL-C and elevated DM. Stratified analysis and interaction tests were used to assess potential variables that may influence the association between TRL-C and elevated DM.

All statistical analyses were performed using the statistical package *R* (http://www.R-project.org) and Empower (R) (http://www.empowerstats.com, X&Y Solutions, Inc., Boston, MA). Statistical significance was defined as two-tailed *P* < 0.05.

## 3. Results

### 3.1. Baseline Characteristics of Participants

**Figure 1:**
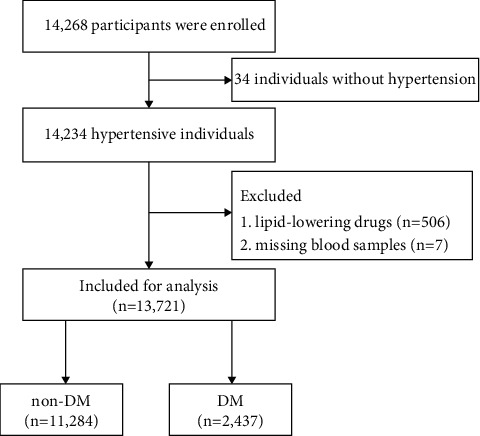
Flow chart of study participants.

After excluding patients taking lipid-lowering drugs and those with missing blood samples, a total of 13,721 hypertensive participants were included in our analysis. The included patients were divided into two groups according to their DM status (Figure 1).  Table 1 shows the baseline characteristics of patients according to the DM status of study participants (mean age: 63.79 ± 9.41 years; 47.22% males). In the study, the prevalence of DM was 17.76% (2,437/13,721), and the average value of TRL-C was 0.61 ± 0.47 mmol/L. The study participants were divided into non-DM and DM groups. Compared with the non-DM group, patients with DM had high values of age, male, BMI, DBP, TC, TG, LDL-C, non-HDL-C, TRL-C, FBG, prevalence of stroke and antihypertensive drugs, and lower values for SBP, current smoking, current drinking, HDL-C, and eGFR (*P* < 0.05).

### 3.2. Crude Associations of DM with Common Risk Factors

Table 2 shows the results of univariate analysis between age, gender, BMI, SBP, DBP, TRL-C, eGFR, current smoking, current drinking, health lifestyle index, history of stroke, history of CAD, and DM, respectively. We found that gender, BMI, SBP, DBP, TRL-C, eGFR, current smoking, current drinking, and history of stroke were significantly associated with DM (*P* < 0.05).

### 3.3. Association of TRL-C with Elevated DM

**Figure 2:**
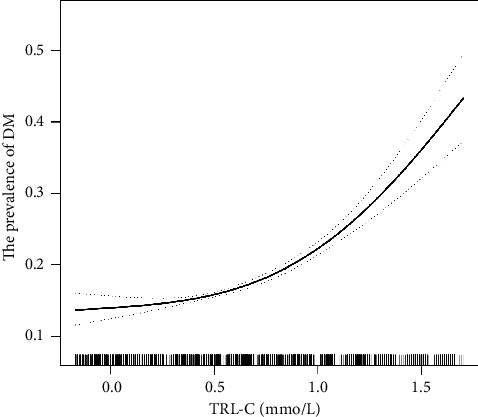
Dose-response relationship between the TRL-C and the prevalence of DM. Adjusted for age, sex, BMI, current smoking, current drinking, SBP, DBP, eGFR, history of stroke and CAD, and antihypertensive drugs.

After adjusting for age, sex, BMI, current smoking, current drinking, SBP, DBP, eGFR, history of stroke and CHD, and antihypertensive drugs, there was a positive association between TRL-C and elevated DM (Figure 2). Per 1 mmol/L increment in TRL-C, the odds ratio (OR) of the risk of elevated DM was 1.73 (95% CI: 1.54, 1.94).

The results of multivariate logistic regression analysis of the relationship between TRL-C and DM were presented by three models (Table 3). Models 1–3 were the crude model, the minor adjusted model, and the fully adjusted model, respectively. The relationship between TRL-C and DM was consistent in the three models. When TRL-C was assessed as quartiles in the fully adjusted model (model 3), the adjusted ORs (95% CIs) for DM in Q2, Q3, and Q4 were 0.94 (0.79, 1.12), 1.21 (1.02, 1.43), and 1.84 (1.57, 2.16), respectively, compared with those in Q1. *P* for trend <0.001 suggesting a dose-response association between TRL-C and elevated DM.

### 3.4. Subgroup Analysis

**Figure 3:**
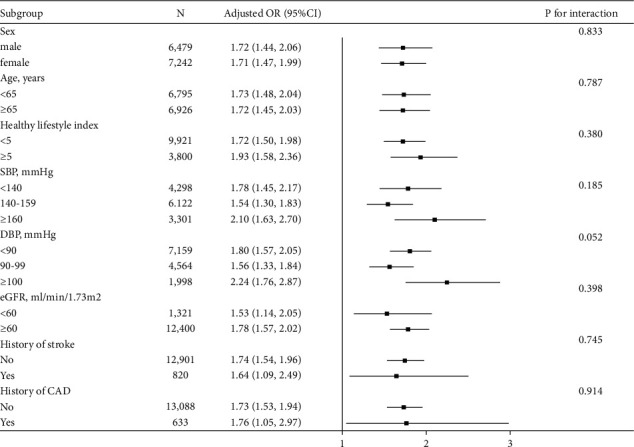
Subgroup analyses of the OR (95% CI) of TRL-C on the prevalence of DM. Adjusted for age, sex, BMI, current smoking, current drinking, SBP, DBP, eGFR, history of stroke and CAD, and antihypertensive drugs, if not be stratified.

Figure 3 shows the relationship between TRL-C and DM in different subgroups. The relationship between TRL-C and DM was positive in the following subgroups: sex (male vs. female; *P*-interaction = 0.833), age (<65 vs. ≥ 65 years; *P*-interaction = 0.787), health lifestyle index (<5 vs. ≥ 5 score; *P*-interaction = 0.380), SBP (<140, 140–159, ≥160 mmHg; *P*-interaction = 0.185), DBP (<90, 90–99, ≥100 mmHg; *P*-interaction = 0.052), eGFR (<60 vs. ≥ 60 mL/min/1.73 m^2^; *P*-interaction = 0.398), history of stroke (no vs. yes; *P*-interaction = 0.745), and history of CAD (no vs. yes; *P*-interaction = 0.914).

## 4. Discussion

In this large-scale population study, the present results showed a positive association between TRL-C and DM. Our findings support TRL-C as a valuable marker for DM. This novel finding expands the application of TRL-C and provides a new therapeutic strategy for patients with diabetes. Moreover, subgroup analysis showed that TRL-C lowering therapy is still necessary for patients with diabetes, even in a healthy lifestyle.

TRL-C, as a novel biological indicator, a series of clinical studies have been carried out around TRL-C. Rosenson et al. [24] found TRL-C as a risk marker for CVD through genetic analysis and suggested that the regulation of the TRL-C gene could reduce CVD risk. Duran et al. [8] followed 976 American healthy women for 15.7 years and found a positive association between TRL-C and CVD, myocardial infarction (MI), and peripheral arterial disease (PAD). Varbo et al. [9] followed 12,512 Danish individuals (general population) for 14 years and found a positive association between TRL-C and ischemic stroke. Cao et al. [25] followed 5,028 Chinese CAD patients for 5.1 years and found that TRL-C was positively associated with prognosis of cardiovascular events. Lamprea-Montealegre et al. [26] followed up 9,270 chronic kidney disease (CKD) patients for 4.9 years and found that TRL-C was significantly associated with CAD risk in CKD patients, and they suggested TRL-C as a new lipid-lowering strategy to reduce CAD risk in CKD patients. Reviewing the abovementioned studies, it is not difficult to find that TRL-C is a reliable indicator of arteriosclerosis-related diseases. In addition, increasing evidence supports that atherosclerosis and the risk of DM are closely related [11, 12, 27]. A recent longitudinal study suggested a causal relationship between atherosclerosis and diabetes, and that atherosclerosis is associated with a higher incidence rate of diabetes [10]. Previous studies have confirmed that TRL-C is a reliable indicator of atherosclerosis. However, to our knowledge, there has not been a study to explore the relationship between TRL-C and DM so far. Our findings demonstrated a positive association between TRL-C and DM, which fills the gaps in diabetes prevention and treatment strategies.

No clear mechanism can fully elucidate the positive association between TRL-C and DM. A reasonable explanation is that TRL-C induces atherosclerosis, which in turn leads to the development of DM. Stage one: TRL-C was deposited in the intima of the artery through transcytosis, which directly led to atherosclerosis [28]. Besides, TRL-C could be directly engulfed by macrophages to form foam cells without oxidative modification, which in turn caused arteriosclerosis [29]. Stage two: arteriosclerosis led to thickening and stiffening of the arterial vessel wall, loss of elasticity, and narrowing of the lumen and caused greater damage to high flow and low-resistance organs, such as the liver and pancreas [30]. When liver function was impaired, liver glycogen synthesis would be weakened, resulting in increased blood glucose [31]. Meanwhile, arteriosclerosis led to decreased blood perfusion in the pancreas, resulting in impaired pancreatic function, resulting in decreased insulin secretion levels and increased blood glucose [32, 33]. In addition, the processes of arteriosclerosis and diabetes are often accompanied by chronic inflammation and oxidative stress [34]. However, further basic experiments are needed to fully elucidate the specific biological mechanism behind this connection.

This study also provides another new insight as a key finding is that the relationship between TRL-C and DM will be undiminished in a healthy lifestyle. A healthy lifestyle, including low BMI, physical exercise, smoking and alcohol cessation, and fresh fruit and vegetable intake, has great benefits for patients with diabetes and prediabetes [13]. The question of whether the association between TRL-C and DM would be weakened in a healthy lifestyle has never been resolved. Our findings suggest that even in a healthy lifestyle, there was still a positive correlation between TRL-C and DM. This finding further supports the importance of TRL-C for the treatment and prevention of diabetes.

The strengths of this study were the inclusion of a large number of hypertensive patients, maximizing adjustment for potential confounders, and aiming to explore the independent relationship between TRL-C and DM in hypertensive patients. In addition, this study examined the positive association between TRL-C and DM in a healthy lifestyle. However, several limitations need to be noted. First, this study was a cross-sectional study and failed to provide a causal relationship between TRL-C and DM. Second, all participants in this study were Chinese hypertensive patients, and further studies are needed to determine whether the findings can be extended to other regions and nonhypertensive patients.

## 5. Conclusion

The present study found an independent positive association between TRL-C and DM in Chinese hypertensive patients. A healthy lifestyle failed to diminish the relationship between TRL-C and DM. Our findings indicate that TRL-C might be a reliable marker of DM and may provide a new strategy for the prevention and treatment of DM.

## Figures and Tables

**Table 1:** Characteristics of study population.

Variables^*∗*^	All patients (*n* = 13,721)	Non-DM (*n* = 11,284)	DM (*n* = 2,437)	*P* value
Age (years)	63.79 ± 9.41	63.56 ± 9.02	64.08 ± 9.87	0.002
Male (*n*) (%)	6,479 (47.22)	2,525 (33.13)	3,954 (64.83)	<0.001
BMI (kg/m^2^)	23.57 ± 3.75	23.25 ± 3.82	23.98 ± 3.61	<0.001
SBP (mmHg)	148.53 ± 17.82	149.45 ± 17.31	147.39 ± 18.39	<0.001
DBP (mmHg)	89.04 ± 10.76	88.75 ± 10.39	89.40 ± 11.20	<0.001
*Physical activity (n) (%)*				
Regular exercise	11,107 (80.95)	9,135 (80.96)	1,972 (80.92)	0.967
Rarely exercise	2,614 (19.05)	2,149 (19.04)	465 (19.08)	

*Fruit and vegetable consumption*				
≥500 g/day	9,204 (82.87)	7,542 (82.56)	1,662 (84.28)	0.066
<500 g/day	1,903 (17.13)	1593 (17.44)	310 (15.72)	

*Smoking status (n) (%)*				
Never	7,959 (58.02)	6,409 (56.81)	1,550 (63.63)	<0.001
Former	2,194 (15.99)	1,815 (16.09)	379 (15.56)	
Current	3,565 (25.99)	3,058 (27.11)	507 (20.81)	

*Drinking status (n) (%)*				
Never	8,900 (64.88)	7,201 (63.83)	1,699 (69.75)	<0.001
Former	1,810 (13.20)	1,511 (13.39)	299 (12.27)	
Current	3,007 (21.92)	2,569 (22.77)	438 (17.98)	

*Laboratory markers*				
TC (mmol/L)	5.18 ± 1.10	5.11 ± 1.05	5.50 ± 1.25	<0.001
TG (mmol/L)	1.80 ± 1.25	1.69 ± 1.12	2.28 ± 1.65	<0.001
LDL-C (mmol/L)	3.00 ± 0.80	2.95 ± 0.77	3.24 ± 0.87	<0.001
HDL-C (mmol/L)	1.57 ± 0.43	1.58 ± 0.42	1.55 ± 0.44	<0.001
Non-HDL-C (mmol/L)	3.61 ± 0.99	3.53 ± 0.95	3.95 ± 1.09	<0.001
TRL-C (mmol/L)	0.61 ± 0.47	0.59 ± 0.45	0.71 ± 0.52	<0.001
FBG (mmol/L)	6.17 ± 1.59	5.68 ± 0.56	8.44 ± 2.55	<0.001
eGFR (ml/min/1.73 m^2^)	88.33 ± 20.19	88.67 ± 19.72	86.75 ± 22.17	0.004
Disease and medication history (*n*) (%)				
Self-reported stroke	820 (5.98)	646 (5.72)	174 (7.14)	0.008
Self-reported CAD	633 (4.61)	506 (4.48)	127 (5.21)	0.121
Antihypertensive drugs	8,784 (64.03)	7,093 (62.87)	1,691 (69.42)	<0.001

Values are presented as mean ± standard deviation, or *n* (%). *DM*, diabetes mellitus; *BMI*, body mass index; *SBP*, systolic blood pressure; *DBP*, diastolic blood pressure; *TC*, total cholesterol; *TG*, triglycerides; *LDL-C*, low-density lipoprotein cholesterol; *HDL-C*, high-density lipoprotein cholesterol; *TRL-C*, triglyceride-rich lipoprotein cholesterol; *FBG*, fasting blood glucose; *eGFR*, estimated glomerular filtration rate; *CVD*, cardiovascular disease.

**Table 2:** Crude association of DM with common risk factors analyzed by univariate analysis.

	Statistics	OR (95% CI)	*P* Value
Age (years)	63.79 ± 9.41	0.99 (0.99, 1.00)	0.024
*Gender*			
Male	6,484 (47.23%)	Ref	
Female	7,244 (52.77%)	1.34 (1.22, 1.46)	<0.001
BMI (kg/m^2^)	23.57 ± 3.75	1.10 (1.09, 1.11)	<0.001
SBP (mmHg)	148.54 ± 17.82	1.00 (1.00, 1.01)	0.020
DBP (mmHg)	89.04 ± 10.76	1.00 (0.99, 1.00)	0.020
TRL-C (mmol/L)	0.61 ± 0.47	1.77 (1.61, 1.94)	<0.001
eGFR (ml/min/1.73 m^2^)	88.33 ± 20.19	1.00 (0.99, 1.00)	<0.001

*Current smoking*			
No	10,156 (74.00%)	Ref	
Yes	3,568 (26.00%)	0.71 (0.64, 0.79)	<0.001

*Current drinking*			
No	10,713 (78.07%)	Ref	
Yes	3,010 (21.93%)	0.74 (0.67, 0.83)	<0.001

*Healthy lifestyle index*			
<5	9,928 (72.32%)	Ref	0.373
≥5	3,800 (27.68%)	0.96 (0.87, 1.06)	

*History of stroke*			
No	12908 (94.03%)		
Yes	820 (5.97%)	1.27 (1.06, 1.51)	0.008

*History of CAD*			
No	13095 (95.39%)		
Yes	633 (4.61%)	1.17 (0.96, 1.43)	0.121

*BMI*, body mass index; *SBP*, systolic blood pressure; *DBP*, diastolic blood pressure; *TC*, total cholesterol; *TG*, triglycerides; *LDL-C*, low-density lipoprotein cholesterol; *HDL-C*, high-density lipoprotein cholesterol; *TRL-C*, triglyceride-rich lipoprotein cholesterol; *eGFR*, estimated glomerular filtration rate.

**Table 3:** Multivariate logistic regression models for prevalence of DM in hypertensive patients.

TRL-C (mmol/L)	Elevated DM OR (95% CI), *P* value					
	Model 1		Model 2		Model 3	
Per 1 mmol/L increase	1.77 (1.61, 1.94)	<0.001	1.72 (1.57, 1.90)	<0.001	1.73 (1.54, 1.94)	<0.001
Quartiles						
Q1 (<0.17)	Ref		Ref		Ref	
Q2 (0.17–0.60)	0.94 (0.82, 1.08)	0.369	0.94 (0.82, 1.07)	0.360	0.94 (0.79, 1.12)	0.472
Q3 (0.60–1.05)	1.23 (1.08, 1.40)	0.002	1.21 (1.06, 1.38)	0.005	1.21 (1.02, 1.43)	0.027
Q4 (≥1.05)	1.95 (1.72, 2.20)	<0.001	1.89 (1.67, 2.14)	<0.001	1.84 (1.57, 2.16)	<0.001
*P* for trend	<0.001		<0.001		<0.001	

Model 1: adjusted for none. Model 2: adjusted for age and sex. Model 3: adjusted for age, sex, BMI, current smoking, current drinking, SBP, DBP, eGFR, history of stroke and CAD, and antihypertensive drugs.

## Data Availability

The datasets used and/or analyzed during the current study are available from the corresponding author on reasonable request.

## Abbreviations

TRL-C: Triglyceride-rich lipoprotein cholesterol
DM: Diabetes mellitus
CVD: Cardiovascular disease
BMI: Body mass index
SBP: Systolic blood pressure
DBP: Diastolic blood pressure
TC: Total cholesterol
TG: Triglyceride
LDL-C: Low-density lipoprotein cholesterol
HDL-C: High-density lipoprotein cholesterol
eGFR: Estimated glomerular filtration rate
FBG: Fasting blood glucose
OR: Odds ratio
CI: Confidence interval.

## Supplementary Materials

Supplementary Materials Table S1: the study inclusion and exclusion criteria. Table S2: components of healthy lifestyle index.
