# Supplementary material for: Positive Association between Triglyceride-Rich Lipoprotein Cholesterol and Diabetes Mellitus in Hypertensive Patients
Source: Int J Endocrinol. 2021 Dec 1;2021:7722269. doi: 10.1155/2021/7722269 (PMC8654545; doi:10.1155/2021/7722269)
Supplement: Supplementary Materials — Table S1: the study inclusion and exclusion criteria. Table S2: components of healthy lifestyle index. [file 7722269.f1.docx]

| Table S1. The study inclusion and exclusion criteria |
| --- |
| Inclusion criteria |
| 1. 18 years of age or older |
| 1. hypertension defined as office systolic blood pressure (SBP) values ≥140 mmHg and/or diastolic BP (DBP) values ≥90 mmHg, self-reported history of hypertension, or the use of antihypertensive drug(s) at baseline |
| 1. signed informed consent |
| Exclusion criteria |
| 1. psychological or nervous system impairment resulting in an inability to demonstrate informed consent |
| 1. unable to be followed up according to the study protocol, or plans to relocate in the near future |
| 1. patients who were not suitable for inclusion or for long-term follow-up as assessed by the study physicians |

| Table S2. Components of healthy lifestyle index. | | |
| --- | --- | --- |
| Healthy lifestyle index components | each factor score 1 | each factor score 0 |
| Body mass index (BMI) | <25 kg/m2 | ≥25 kg/m2 |
| Physical activity | ≥150 minutes/week of moderate intensity physical activity or ≥75 minutes of vigorous-intensity physical activity | <150 minutes/week of moderate-intensity physical activity and <75 minutes of vigorous intensity physical activity |
| Smoking status | Non-smoking (never or former) | Current smoking |
| Drinking status | Non-drinking (never or former) | Current drinking |
| Fruit and vegetable consumption | Fresh fruit and vegetable consumption ≥500g/day | Fresh fruit and vegetable consumption <500g/day |
